# Knowledge, attitude, and practice toward foodborne disease among Chinese college students: a cross-sectional survey

**DOI:** 10.3389/fpubh.2024.1435486

**Published:** 2024-12-17

**Authors:** Xingming Ma, Li Bo, Xinmiao Zhou

**Affiliations:** ^1^School of Health Management, Xihua University, Chengdu, China; ^2^School of Food and Biological Engineering, Xihua University, Chengdu, China

**Keywords:** foodborne diseases, China, knowledge, attitudes, practices, students

## Abstract

**Background:**

More than 200 diseases are caused by eating food contaminated and the burden of foodborne disease is considered to be high worldwide. Foodborne diseases are an important public health issue, and the knowledge, attitudes and practices among college students are crucial in preventing outbreaks.

**Objective:**

This study aimed to evaluate the current knowledge, attitudes, and practices (KAP) toward foodborne diseases among Chinese college students and to identify the factors influencing practice actions.

**Methods:**

A cross-sectional study was conducted from April to November in 2023. A cluster random sampling method was used to enroll participants at Xihua University. The questionnaire including 47 items was used to collect the foodborne diseases information. An offline questionnaire was used to generate the surveys. Multiple linear regression was used to analyze the factors associated with foodborne disease knowledge, attitudes and practices.

**Results:**

A total of 445 college students with a mean age of 19.76 ± 1.24 years were included in the study. The majority of participants were female (59.1%), of Han Chinese (95.1%), and nearly two-thirds were lower-year undergraduates (66.7%). Most participants (78.9%) were non-medical students, and 64.7% of participants were from rural areas. The KAP score of foodborne diseases was 79.21 ± 6.84. The knowledge, attitudes, and practices scores toward foodborne diseases were 8.76 ± 0.95, 10.50 ± 1.63, and 59.94 ± 6.48, respectively. In particular, there was a notable decrease in practice scores toward foodborne diseases among male students, ethnic minority students, and non-medical students. The significant predictors of practices were included gender (*β* = 0.11, *p* < 0.05), nationality (*β* = −0.12, *p* < 0.05), specialization type (*β* = −0.13, *p* < 0.05), residence (*β* = −0.11, *p* < 0.05).

**Conclusion:**

These results revealed gaps in knowledge, attitudes, and practices regarding foodborne diseases, and the knowledge was at an insufficient level, the attitude was positive and practice was at a moderate and acceptable level. In general, the level of KAP was at a moderate and acceptable level. Gender, nationality, education level, specialization type, and residence were identified as crucial influencers on practices toward foodborne diseases. It is necessary to provide foodborne disease health education targeting this population in western areas, which can help improve students’ knowledge, attitudes, and practices.

## Introduction

Foodborne diseases (FBDs) are infections of the digestive system caused by foodborne pathogens such as parasites, viruses, bacteria, and fungi, which are a public health threat, leading to loss of lives and hindering socioeconomic development (1, 2). Over 250 different types of microbial agents and unconventional agents such as prions are linked to foodborne illnesses in humans (3). Microbial infections were the most common cause of outbreaks worldwide. In particular, bacteria such as Salmonella, Shigella species, pathogenic *Escherichia coli*, and *Listeria monocytogenes* are the most commonly infection sources of foodborne disease (4). Those agents of foodborne disease are from cross-contamination or contaminated food including poultry, eggs, meat, vegetables and ready-to-eat foods, and can affect individuals of all ages (5, 6).

It was estimated that unsafe foods led to 600 million cases of foodborne illness, 420,000 deaths, and the loss of 33 million years of healthy life globally (7). In the United States, approximately100,939 cases of foodborne illnesses, 5,699 hospitalizations, and 145 deaths were reported between 2009 and 2015, and the proportion of foodborne illnesses attributed to food-producing animals was 10.4–14.1% from 1999 to 2017 (8, 9). In Europe, 23 million people are affected by foodborne illnesses, leading to approximately 5,000 deaths annually (8). According to the data from the China National Foodborne Disease Outbreaks Surveillance System, a total of 5,493 outbreaks of foodborne disease were reported in 2021, resulting in 117 deaths and 32,334 illnesses from 30 provinces, and the incidence rate of foodborne diseases is 2.43 per one hundred thousand (10).

The location where food raw materials and its products were contaminated, allowing pathogens to survive and multiply, varied in each outbreak. Food raw materials and their products are contaminated during processing, preparation, preservation, and consumption, resulting in an outbreak of foodborne diseases. A study showed that foodborne disease outbreaks in China occurred mainly in food service establishments, homes and schools in 2021 (10). Although the majority outbreaks of foodborne-disease are caused by various food service facility providers, schools and homes are important sites where foodborne diseases have been reported to occur frequently and can affect students of all ages. In the post-COVID-19 pandemic era, takeaway food services are an emerging business in China and have become one of the potential options for college students’ campus foods (11). Notably, the takeaway ordering services and food delivery around the school are gaining popularity among students due to their affordability, convenience, and easy accessibility from takeaway platforms, attracting them as a preferred meal option for college students (12). For college students, while it is convenient to eat takeaway foods, there is also a potential impact on both college student health and campus food safety (13, 14).

The majority of foodborne diseases reported cases occur in commercial food service institutions, so food consumers and handlers play a fundamental role in preventing foodborne diseases. Most instances of foodborne disease can be prevented if food consumers and handlers follow the principles of food safety (15, 16). Numerous studies have been focused on the impact assessment of various education and training interventions on the food safety knowledge, attitudes, and actions of food handlers in food service establishments. A meta-analysis of 18 randomized controlled trials and 29 nonrandomized trials has shown that food safety education and training interventions are effective strategies for improving the behaviors and behavioral precursors (e.g., knowledge and attitudes) of food handlers (17).

However, consumer surveys revealed different levels of understanding of foodborne diseases, and the application of this knowledge was also found to be inconsistent. Studies on consumers have shown that foodborne diseases outbroke, attributing to improper food preparation practices, decreased personal hygiene, and a lack of food safety knowledge among consumers (18, 19). In China, college students are a special group of people who are usual both food preparation practices and consumers at school. Common improper practices include long-term storage at room temperature for consumption, cross-contamination of raw ingredients, inadequate cold-holding temperatures, insufficient time and temperature during reheating, inadequate cleaning of utensils, and using of undercooked foods etc. (9, 20, 21).

Student knowledge and practices related to food safety can play a fundamental role in the prevention of foodborne diseases (22). In order to enhance the oversight and control of foodborne disease, the Chinese government enacted the food safety law in 2009, with the second amendment taking place in 2021 (23). Subsequently, the Ministry of Education of the People’s Republic of China, the State Administration for Market Regulation of the People’s Republic of China, and the National Health Commission of the People’s Republic of China all together promulgated regulations on the management of food safety and nutrition and health in schools in 2019 (24).

It is stipulated that the school food processing practices must comply with the school’s food safety and nutrition health management regulations. It should guide college students in developing healthy eating habits and good personal hygiene, as well as to improve their awareness and knowledge of food safety, with which is to reduce the risk of food contamination and reduce the occurrence of foodborne illness for themselves (23, 24). Despite a series of action plans in the food safety field including school-food safety in China, the knowledge and practices of students regarding foodborne diseases and food safety were found unsatisfactory. Especially in western China, there was no significant decrease in the overall reporting of foodborne illness among school students (25).

Therefore, it is crucial to evaluate college students’ knowledge, attitudes, and practices toward foodborne diseases in order to find ways to reduce the risk of foodborne diseases. However, most foodborne illness studies in China have focused on epidemiology (26, 27), public food establishments (28, 29), investigation of middle schools in rural areas (30), without addressing the knowledge, attitudes, and practices among students in higher education in recent years. In China, the article on foodborne illness studies among the college students was limited to the Henan in 2015 and Chongqing in 2019. In the post-COVID-19 pandemic era, the eating habits of young people have changed significantly, and they are paying more attention to healthy living and food safety. While this study did not obtain a nationally representative sample, it still offered insight into the knowledge, attitudes, and practices of college students in western China, and contributed important data to regional studies in the area in the post-pandemic. This study used the knowledge, attitudes, and practices (KAP) tool to assess the KAP level toward foodborne diseases among college students in the western of China, and to identify the factors that influence practice actions.

## Materials and methods

### Study design and setting

In order to evaluate students’ knowledge, attitudes, and practices (KAP) concerning foodborne diseases, a cross-sectional survey was conducted at Xihua University in China from the spring semester to the fall semester, which took place between April and November 2023. The report of this study followed the STROBE checklist for observational studies (Supplementary Table S1).

A cluster random sampling method was used to enroll participants at our university, a public research university with a population of ~41,000 undergraduate students located in Chengdu. Three classes of students from general education courses were randomly selected and included in the study. Then all students in each selected class from freshmen to seniors were included to participate in the study.

Seniors are fourth-year students, juniors are third-year students, sophomores are second-year students, and freshmen are first-year students in university. Freshmen and sophomores are the lower-year undergraduates; juniors and seniors are the upper-year undergraduates. An offline questionnaire was used to generate the surveys. The survey took about 5–8 min to complete, and students who answered fewer than 95% of the questions were excluded from the study.

### The inclusion and exclusion criteria of participants

The inclusion criteria for the study were as follows: (a) voluntary participation of all students (b) un-occurred of foodborne diseases in the past 2 months, (c) and no limited to grades and majors including medical students and non-medical students. The exclusion criteria were as follows: (a) unwillingness to participate in the study and (b) the occurrence of foodborne diseases in the past 2 months.

### Sample size calculation

A sample size of participants was calculated using Cochran’s formula (31), N = Z^2^*p*q/*e*^2^. Test significance level was set *α* = 0.05, *e* is the margin of error at 5% (standard deviation of 0.05), Z is 1.96 at 95% confidence interval, and q = 1 − *p*. According to previous studies on this topic have been conducted in China, the anticipated frequency *p* has been established at 50% (32). In this study, a minimum sample size of participants was 384. Considering potential dropouts, 450 students were selected for the study.

### Ethics

The current study was conducted in accordance with the guidelines of the Declaration of Helsinki. We provided assurances regarding respondents’ confidentiality and emphasized the study’s voluntary nature. Those participants were assured that the data would be used only for research purposes and informed consent was obtained from all participants (Supplementary Table S2).

### Questionnaire design

A questionnaire used to gather data on KAP was based on a literature review of previous studies (33–35). The questionnaire was presented in a Chinese version and then the content validity was evaluated by a microbiologist and two public health experts from the university. The questionnaire included all 47 items, which were divided into four domains (36) (Supplementary Table S3).

The first domain with 9 questions collected sociodemographic data from participants. The sociodemographic variables included gender, age, nationality, educational level, monthly living expenses, specialization type, residence, training on foodborne diseases, and onset within 2 months. The second domain with 10 items assessed participants’ knowledges toward foodborne diseases, which included the knowledges of foodborne diseases, food contamination pathways and control. The knowledge section of the questionnaire consisted of “true or false” questions for each item, and a correct response got 1 point for a total of 10 points. The scores of participants who correctly answered the questions were calculated in the knowledge domain. The third domain with 13 items evaluated participants’ attitudes toward foodborne diseases in the three-point scale pattern, ranging from agree (positive) to disagree (negative), and a positive attitude got 1 point for a total of 13 points. The scores of participants who positively answered the questions were calculated in this domain. In the fourth domain, a five-point scales with 15 questions was used to assess participants’ practices toward foodborne diseases. This section required respondents to indicate whether they never, sometimes, neutral, often, or always engaged in specific behaviors. The score ranged from one (never), two (sometimes), three (neutral), four (often) to five (always) for items of 1, 2, 3, 4, 9, 12, 13, 14, 15, while the score assigned 5 to never, 4 to sometimes, 3 to neutral, 2 to often, and 1 to always for items of 5, 6, 7, 8, 10, 11, and then total practices scores were calculated.

### Data analysis

IBM SPSS Statistics version 22 (IBM Corp., Armonk, NY, United States) was used to analyze the data. All data of students’ KAP (knowledge, attitudes, and practices) scores were presented as means and standard deviations (SD). The knowledges content and validity were confirmed by experts in their corresponding fields. Cronbach’s *α* was utilized to assess the internal consistency (i.e., reliability), the Kaiser-Meyer-Olkin coefficient and Bartlett’s test of sphericity were used to assess the construct validity of the questionnaires (attitudes and practices domains), 0.6 ~ 0.7 or higher is acceptable level (37, 38) and statistical significance was set at *p* < 0.05. The Chi-square test was used to analyze the differences in categorical variables, and the independent sample *T*-test and one-way analysis of variance (ANOVA) were used to analyze the differences in continuous variables among demographic characteristics. Multiple linear regression was utilized to determine the factors linked to knowledge, attitudes, and practices scores regarding foodborne disease. Regression coefficients (*β*) with 95% CI were employed to measure these associations.

## Results

### Participants demographic characteristics

A total of 448 (the response rate of 97%) students participated in the study (Table 1). After excluding 3 incomplete questionnaires, 445 questionnaires were included, resulting in an effective recovery rate of 99.3%. The majority of the participants were female (59.1%) and Han Chinese (95.1%). The age of the participants was between 18 and 24 years. Almost two-thirds of the participants (66.7%) were the lower-year undergraduates (freshman and sophomore), and 33.3% were the upper-year undergraduates (junior and senior). Almost four-fives of participants’ (87%) monthly living expenses were ranging from 1,000 Yuan to 2,000 Yuan. Most of participants (78.9%) were non-medical students and only 21.1% were medical students. Around 35.3% of the participants were from cities or towns and 64.7% of the participants were from rural area. A majority (91.8%) of participants did not receive any training on foodborne diseases and/or food safety, and only 9.2% of the participants completed any food safety training or courses. All students did not have a foodborne illness in 2 months (Table 1).

**Table 1:** Demographic characteristics and KAP score analysis of the participants.

Variables	Proportion (*N* = 445)	Frequency (%)	Scores of KAP (mean ± SD)	Statistical value
Gender				
Male	182	40.9%	78.47 ± 7.41	*t* = 1.32, *p* = 0.18
Female	263	59.1%	79.72 ± 6.37	
Age, years				
18 ~ 20 years	313	70.3%	79.38 ± 6.58	*t* = 0.01, *p* = 0.99
≥20 years	132	29.7%	78.81 ± 7.41	
Nationality				
Ethnic minority	22	4.9%	75.63 ± 6.09	*t* = −2.53, *p* = 0.01
Han Chinese	423	95.1%	79.39 ± 6.83	
Educational level				
Lower-year undergraduate	297	66.7%	78.75 ± 6.80	*t* = −2.01, *p* = 0.04
Upper-year undergraduates	148	33.3%	80.13 ± 6.83	
Monthly living expenses				
<1,000 yuan	16	3.6%	77.50 ± 4.95	*F* = 6.90, *p* = 0.001
1,000 ~ 2000 yuan	387	87.0%	78.89 ± 6.71	
>2000 yuan	42	9.4%	82.80 ± 7.57	
Type of specializations				
Non-medical field	351	78.9%	78.64 ± 6.86	*t* = −3.43, *p* = 0.001
Medical field	94	21.1%	81.34 ± 6.34	
Residence				
Cities and towns	157	35.3%	80.62 ± 7.10	*t* = 3.24, *p* = 0.001
Rural area	288	64.7%	78.44 ± 6.57	
Training on foodborne diseases				
Yes	41	9.2%	82.48 ± 6.04	*t* = 3.25, *p* = 0.001
No	404	90.8%	78.88 ± 6.83	
Onset within 2 months				
Yes	0	0	–	
No	445	100%	78.79 ± 6.63	–

Scores of knowledge, attitudes and practices (KAP) were calculated. Independent sample *t*-test and one-way ANOVA test used for KAP scores toward foodborne diseases. Lower-year undergraduate (freshman + sophomore) and upper-year undergraduate (junior and senior).

Cronbach’s alpha coefficient in this study was 0.61 and 0.65 for students’ attitudes and practices toward foodborne diseases respectively, which was acceptable level. The Kaiser-Meyer-Olkin coefficient was 0.67 for students’ attitudes and Bartlett’s test of sphericity was statistically significant (*p* < 0.001, χ^2^ = 646.39, and df. = 78), and the Kaiser-Meyer-Olkin coefficient was 0.70 for students’ practices and Bartlett’s test of sphericity was statistically significant (*p* < 0.001, χ^2^ = 1403.76, and df. = 105), indicating the acceptable validity of the scale section. The mean score of foodborne diseases KAP was 79.21 ± 6.84 out of 98 (range = 60 to 96). Of the 445 students, 234 students (52.6%) had a high KAP level and scored over 80 points toward foodborne diseases. The study findings showed a significant difference in foodborne diseases KAP score based on the nationality, educational level, monthly living expenses, specialization type, residence, and training on food safety (all, *p* < 0.05) (Table 1).

### Knowledge toward foodborne diseases

**Figure 1:**
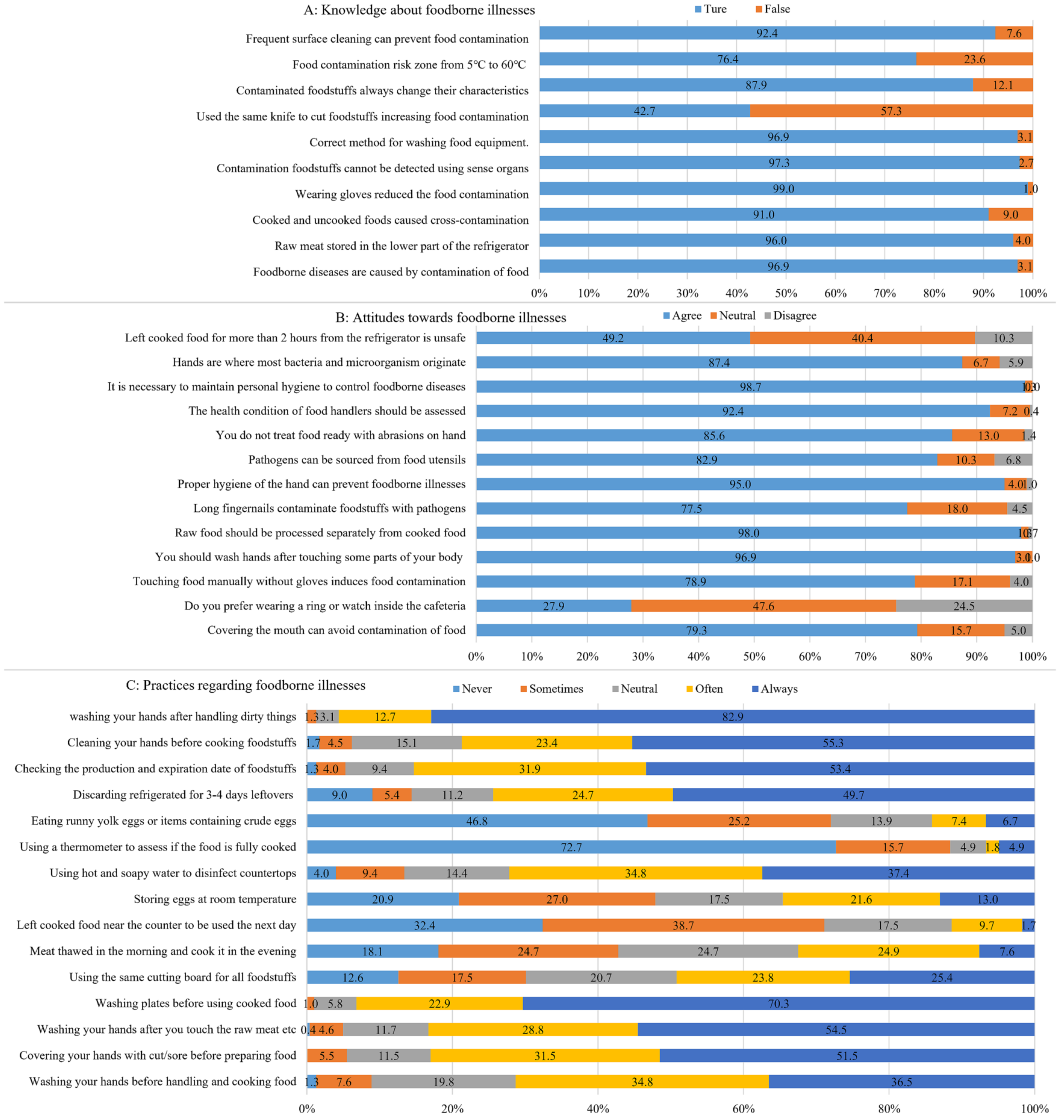
Responses to questions related to KAP (knowledge, attitude, and practice) toward foodborne diseases (*n* = 445). **(A)** Knowledge about foodborne illnesses; **(B)** Attitude toward foodborne illnesses; **(C)** Practice regarding foodborne illnesses.

An assessment of foodborne diseases knowledge of students is summarized in Table 2. 80% ~ 89% of the students were able to accurately comprehend foodborne diseases-related knowledge (Supplementary Table S4). Out of 10 foodborne diseases-related questions, a large proportion (89.3%) of the college students can answer correctly about 7 questions. The mean score of foodborne diseases knowledge was 8.76 ± 0.95 ranging 6 to 10. Of the 455 participants, almost two-thirds of students (65.2%, *n* = 290) had a moderate knowledge level with scores >80%. Most of the participants showed moderate knowledge of foodborne diseases including food contamination pathways and control ways, personal hygiene and habits. But poor knowledge was mainly related to cross-contaminating food with the same knife to cut raw meat and vegetables (Figure 1).

**Table 2:** The scores of foodborne diseases-related knowledge, attitudes and practices among participants.

Variables	Proportion (*N* = 445)	Knowledge scores (mean ± SD)	*p*-value	Attitudes scores (mean ± SD)	*p*-value	Practices scores (mean ± SD)	*p*-value
Gender							
Male	182	8.84 ± 0.89	0.19	10.56 ± 1.71	0.54	59.08 ± 6.93	**0.02**
Female	263	8.71 ± 0.99		10.46 ± 1.58		60.54 ± 6.09	
Age, year							
<20	313	8.73 ± 0.96	0.32	10.44 ± 1.73	0.17	59.61 ± 6.05	0.19
≥20	132	8.83 ± 0.95		10.65 ± 1.37		59.30 ± 6.85	
Nationality							
Ethnic minority	22	8.91 ± 0.81	0.40	10.81 ± 1.13	0.35	55.91 ± 7.00	**0.003**
Han nationality	423	8.76 ± 0.96		10.48 ± 1.66		60.15 ± 6.83	
Educational level							
Freshman+ Sophomore	297	8.67 ± 0.95	**0.005**	10.40 ± 1.77	**0.046**	59.27 ± 6.35	0.21
Junior+ Senior	148	8.95 ± 0.94		10.70 ± 1.31		60.02 ± 6.17	
Monthly living expenses							
<1,000 yuan	16	8.75 ± 1.00	0.12	10.37 ± 1.45	**0.009**	58.37 ± 5.41	**0.003**
1,000 ~ 2000 yuan	387	8.80 ± 0.96		10.42 ± 1.67		59.66 ± 6.35	
>2000 yuan	42	8.48 ± 0.86		10.23 ± 1.12		63.09 ± 7.24	
Type of specializations							
Non-medical field	351	8.73 ± 0.94	0.14	10.42 ± 1.72	**0.015**	59.49 ± 6.86	**0.004**
Medical field	94	8.89 ± 1.02		10.80 ± 1.23		61.63 ± 6.34	
Residence							
Cities and towns	157	8.62 ± 1.04	**0.029**	10.62 ± 1.51	0.25	61.37 ± 6.74	**0.001**
Rural area	288	8.84 ± 0.90		10.43 ± 1.70		59.16 ± 6.24	
Training on foodborne diseases							
Yes	41	8.88 ± 0.90	0.42	10.82 ± 1.33	0.18	62.78 ± 5.59	**0.003**
No	404	8.75 ± 0.96		10.47 ± 1.66		59.65 ± 6.50	

Bold indicates significance at *p* < 0.05, Scores of knowledge, attitudes and practices were calculated, respectively. Independent sample *t*-test and one-way ANOVA test used for knowledge, attitudes and practices scores toward foodborne diseases. For knowledge toward foodborne diseases, each correct answer that was addressed as “True” was marked as 1 point, while “False” responses were given 0 points in this section. For attitudes toward foodborne diseases, each positive response that was addressed as “agree” was marked as 1 point, while “neutral” and “disagree” responses were given 0 points in this section. For practices toward foodborne diseases, 1 = never, 2 = sometimes, 3 = neutral, 4 = often, 5 = always for items 1,2,3,4,9,12,13,14,15 and 5 = never, 4 = sometimes, 3 = neutral, 2 = often, 1 = always for item 5,6,7,8,10,11.

In the demographic characteristics variable comparison, the mean score of foodborne diseases knowledge in the upperclassman (junior/senior) (8.95 ± 0.94) was visible higher than that of the underclassmen (freshman/sophomore) (8.67 ± 0.95) (*p* < 0.05). The mean score of foodborne diseases knowledge in students from the rural areas (8.84 ± 0.90) was noticeable higher than that of students form the cities and towns (8.62 ± 1.04) (*p* < 0.05). However, there was no statistical difference in students’ knowledge toward foodborne diseases in the variables of gender, age, nationality, monthly living expenses, specialization type and training on food safety (all, *p* > 0.05) (Table 2).

### Attitudes toward foodborne diseases

Most of the students (78% ~ 86%) showed positive attitudes toward foodborne diseases (Supplementary Table S5). On a 3-point scale, the overall average score of the questions on attitudes was 10.50 ± 1.63 out of 13, ranging 1 to 13. Among the study participants, 4.9% responded negatively, 14.3% were neutral, and 80.8% had positive attitudes toward foodborne diseases. Only 27.9% of participants believed that wearing a ring or watch would cause food contamination inside the cafeteria. Nearly half of the respondents (49.2%) agreed that leaving cooked food out of the refrigerator for more than 2 h was unsafe. Approximately 78.9% of participants believed that touching food manually without gloves would induce a cross-contamination of foods, and 77.5% of participants expressed that pathogens such as bacteria could contaminate food through long and painted fingernails (Figure 1).

In the demographic characteristics variable comparison, the mean score of attitudes toward foodborne diseases in the upperclassman (junior/senior) (10.7 ± 1.31) was significantly higher than that of the underclassmen (freshman/sophomore) (10.4 ± 1.77) (*p* < 0.05). The average score for attitudes toward foodborne diseases among medical students (10.80 ± 1.23) was significantly higher than that of non-medical students (10.42 ± 1.72) (*p* < 0.05). The attitudes of students with a monthly living expense of 1,000–2000 toward foodborne diseases are more positive, with the highest significant score (10.42 ± 1.67) (*p* < 0.05). There was no statistical difference in students’ attitudes toward foodborne diseases in the variables of gender, age, nationality, residence and training on food safety (all, *p* > 0.05) (Table 3).

**Table 3:** The multiple linear regression of knowledge, attitudes, and practices scores toward foodborne diseases.

Variables	Knowledge level				Attitudes level				Practices level			
	*β*	*p*-value	95%CI		*β*	*p*-value	95%CI		*β*	*p*-value	95%CI	
			Lower	Upper			Lower	Upper			Lower	Upper
Gender												
Male	RC											
Female	−0.07	0.16	−0.31	0.05	0.01	0.99	−0.30	0.32	0.11	**0.024**	0.18	**2.58**
Age, year												
<20	RC											
≥20	−0.08	0.20	−0.43	0.09	0.01	0.90	−0.41	0.47	−0.14	0.26	−3.61	−0.24
Nationality												
Ethnic minority	0.04	0.40	−0.24	0.59	0.04	0.40	−0.41	1.00	−0.12	**0.012**	−6.15	−0.77
Han nationality	RC											
Educational level												
Freshman+ Sophomore	RC											
Junior+ Senior	0.19	**0.008**	0.10	0.67	0.01	0.85	−0.44	0.53	0.08	0.25	−0.79	2.94
Monthly living expenses												
<1,000 yuan	RC											
1,000 ~ 2000 yuan	0.02	0.86	−0.44	0.53	−0.01	0.86	−0.90	0.76	0.02	0.86	−2.88	3.45
>2000 yuan	−0.09	0.29	−0.87	0.26	0.13	0.13	−0.22	1.70	0.17	0.05	0.05	7.43
Type of specializations												
Non-medical field	0.01	0.89	−0.25	0.29	−0.07	0.25	−0.73	0.19	−0.13	**0.024**	−3.82	−0.27
Medical field	RC											
Residence												
Cities and towns	RC											
Rural area	0.09	0.07	−0.02	0.36	−0.06	0.23	−0.52	0.13	−0.11	**0.015**	−2.80	−0.30
Training on foodborne diseases												
Yes	0.04	0.35	−0.16	0.45	0.06	0.20	−0.19	0.86	0.11	**0.013**	0.53	4.54
No	RC											
Knowledge score	–				0.15	**0.002**	0.10	0.42	−0.04	0.43	−0.87	0.37
Attitudes score	–				–				0.03	0.52	−0.24	0.48
Practices score	–				–				–			

Bold indicates significance at *p* < 0.05. “–“Not included. The *R*^2^ for the model of knowledge score, attitudes score and practice score were 0.05 (*F* = 2.42, *p* = 0.011), 0.06 (*F* = 2.71, *p* = 0.003), and 0.12 (*F* = 5.45, *p* < 0.001), respectively. *β*, Regression coefficient; CI, confidence interval; RC, reference category.

### Practices toward foodborne diseases

Less than half of the students (35% ~ 54%, always) had varied adequate practice, and about one-thirds of the students had inadequate practice toward foodborne diseases (Supplementary Table S6). The mean score of the practices toward foodborne diseases was 59.94 ± 6.48 out of 75, ranging from 41 to 75. Of the 445 participants, slightly more than half (52%, *n* = 230) of students had a better level of practices toward foodborne diseases with scores >80%. As shown in Figure 1, almost 82.9% of the students wash hands after handling dirty things. Before serving cooked food, 70.3% of the students wash bowls and plates used for seafood, poultry and raw meat. Nearly half of the students never eat runny yolk eggs or items containing crude eggs (46.8%), and always discard refrigerated leftovers after 3–4 days (49.7%). About 53.4% of the students always check the production date and expiration date and never ate food that deteriorated and expired. However, one quarter of students (25.4%) always use the same cutting board for raw meat, poultry, seafood and vegetables, and 13.0% of the students always long store eggs at room temperature.

In the demographic characteristics variable comparison, the average score of practices toward foodborne diseases was significantly varied by participants’ gender, nationality, monthly living expenses, specialization type, residence and training on food safety (all, *p* < 0.05) (Table 2). Male students (59.08 ± 6.93), students from ethnic minorities (55.91 ± 7.00), students with low monthly living expenses (58.37 ± 5.41), non-medical students (59.49 ± 6.86), students from rural areas (59.16 ± 6.24), and students without food safety training, those scored lower in the practices toward foodborne diseases. However, there was no significant difference in students’ practices toward foodborne diseases in the variables of age and educational level (all, *p* > 0.05) (Table 2).

### Assessment of the variables associated

The multiple linear regression of knowledge, attitudes, and practices scores toward foodborne diseases was showed in Table 3. Data of Pearson correlation showed that students’ attitudes regarding foodborne diseases improved as their knowledges of foodborne diseases increased (*p* < 0.05). The data of regression indicated that knowledge of participants was positively associated with their attitudes (*β* = 0.15, *p* < 0.05), but not with their practices (*β* = −0.04, *p* > 0.05). The regression models showed that upperclassmen (junior and senior) had high knowledge levels on foodborne diseases compared to the underclassmen (freshman and sophomore) (*β* = 0.19, *p* < 0.05), which showed that educational level was the significant predictor of knowledge on foodborne diseases. One meaningful factor of attitudes toward foodborne diseases was the knowledge (*β* = 0.15, *p* < 0.05). Students with high knowledge levels were appreciably more likely to have a positive attitude toward foodborne diseases. On the other hand, the significant predictors of practices were included gender (*β* = 0.11, *p* < 0.05), nationality (*β* = −0.12, *p* < 0.05), specialization type (*β* = −0.13, *p* < 0.05), residence (*β* = −0.11, *p* < 0.05), and training on foodborne diseases (*β* = 0.11, *p* < 0.05).

## Discussion

Foodborne illnesses continue to be a major public health issue in developing countries (39), and understanding the knowledge, attitudes, and practices (KAP) of individuals, particularly college students, is also crucial for preventing foodborne illnesses. The present study provided valuable insights into the knowledge, attitudes, and practices of foodborne diseases among college students. Overall, this study found a moderate KAP score level among college students in western areas.

The role of college student food consumers plays a crucial part in reducing foodborne diseases. Previous studies have indicated that health literacy levels could effectively assist individuals in preventing and controlling disease (40). Students with adequate health literacy may have higher KAP scores for foodborne diseases, and were more likely to be aware of their responsibility to prevent foodborne diseases and then to adopt beneficial health practices. Students with low health literacy may lack access to valuable health knowledge and struggle to adopt beneficial health practices (41). Although China has made considerable progress in improving health literacy in the last decades (42), foodborne disease health education for college students remains an important means for improving population health. In the study, the identification of only 47.4% of students with a low KAP level suggests that there is still a significant portion of the students that may benefit from targeted educational interventions to improve their understanding and practices related to foodborne diseases.

The study results revealed that participants with differences in nationality, educational level, specialization type, and residence had a substantial variation in overall KAP scores for foodborne diseases (*p* < 0.05). There was a meaningful decline in the KAP score toward foodborne diseases among ethnic minority students, freshman and sophomore students, non-medical students, rural students and students who were not trained in foodborne diseases. However, the findings for demographic characteristics variables are not fully consistent with previous studies in Jordan (43), Greek (44), Kuwait (45), Canadian (46), Bulgaria (47), Saudi Arabia (48), and Ethiopia (33), which may be attributed to differences in study design, questionnaire format, study period, social support, and demographic characteristics. Overall, this suggests that interventions and educational programs targeting foodborne diseases should be tailored to address the specific needs and characteristics of these younger cohorts. Efforts to enhance and promote foodborne diseases training programs are crucial in equipping college students with the necessary knowledge and skills to mitigate foodborne disease risks.

The study found that the participants displayed a moderate level of knowledge regarding foodborne diseases, with the majority answering the knowledge-related questions correctly. The average knowledge score of foodborne diseases varied significantly across different sociodemographic characteristics, consistent with findings in other studies (46–48) on food safety knowledge. However, there were specific areas of poor knowledge, particularly concerning practices such as cross-contaminating food with the same knife to cut raw meat and vegetables, and long-storing eggs at room temperature. Additionally, the study revealed that upperclassmen (junior and senior students) had a higher knowledge level on foodborne diseases compared to the underclassmen (freshmen and sophomore students), indicating that educational level was a significant predictor of knowledge on foodborne diseases.

Attitude is an important factor that can influence perceptions and practices related to foodborne diseases, ultimately leading to a decrease in the occurrence of foodborne illnesses (49, 50). The results showed that students with a high level of knowledge about foodborne diseases were appreciably more likely to have a positive attitude toward foodborne diseases practices. Based on the attitude of all questions, 81% of college students had a positive response toward foodborne diseases, higher than previously estimated levels from an evaluation of food safety education on college students in Henan Province, China, in 2014 (51). This difference might be due to the implementation of regulations on the management of food safety, and nutrition and health in schools in 2019 (24) and improvement in health literacy in the last decades in China (42). This finding underscores the importance of knowledge in shaping individuals’ attitudes and perceptions toward foodborne diseases. It also highlights the potential for targeted educational interventions to improve attitudes and promote safer food practices among college students.

In the current study, there was a moderate level of practices toward foodborne diseases among college students, which aligns with several previous studies in Jordan (52) and Bangladesh (53). Additionally, the study also revealed that individuals with higher monthly living expenses were more inclined to adhere to positive attitudes and good food hygiene practices. Most students showed good habits of avoiding food kept at room temperature for a long time and refusing to eat expired food, which is in line with a prior survey conducted among students from China Chongqing’s nursing, education, and medical colleges (32). However, there were areas for improvement, for example, one quarter of students reported practices such as using the same cutting board for vegetables, seafood, and raw meat, and long storing eggs at room temperature, which are known foodborne diseases risks. In particular, male students, ethnic minority students, non-medical students, and students from rural areas showed a lower score in the practices toward foodborne diseases, which suggests the need for targeted interventions to improve specific foodborne diseases practices among college students.

Multiple linear regression analyses (Table 3) showed that gender, nationality, education level, specialization type, residence and training on foodborne diseases were identified as key influencers on foodborne diseases practices, which are consistent with previous studies in China (32), Saudi Arabia (48), Ethiopia (33) and Bangladesh (54). The study found that knowledge of foodborne diseases provokes positively attitudes toward foodborne diseases, consistent with a study on Bangladesh students (47). This underscores the importance of considering these factors when developing interventions to improve foodborne diseases practices among Chinese college students. Targeted educational programs that address specific knowledge gaps and promote positive attitudes toward foodborne diseases practices could be instrumental in improving overall food safety behaviors among this population.

In general, although the participants had a positive attitude, their practice was still sometimes lower than expected. Students should be aware of the importance of good practices in preventing foodborne illness. For example, when buying food, check the production date and shelf life. Wash hands before handling or cooking food and after contact with raw meat, poultry, seafood, or contaminated items. Discard leftovers after 3–4 days in the refrigerator. Use separate cutting boards for raw meat, poultry, seafood, and vegetables. Use clean plates for cooked foods and do not mix raw and cooked foods. Bandage any cuts on your hands before preparing ingredients, etc.

Nonetheless, this study has several limitations. Firstly, the selection of universities utilized a non-probability sampling technique, which could potentially bias the study’s findings. Secondly, the sample was obtained from only one university, with a limited number of college students included in the study. Thirdly, the ethnic minority sample used in this study was significantly smaller compared to the Han nationality group, and a more diverse ethnic minority sample should be included in future research. Fourthly, it employed a cross-sectional design, and thereby could not claim causality among the variables studied. Furthermore, given the self-report nature of the measurements employed in this study, there is a risk of social desirability and reporting biases from the respondents. Due to these limitations, the findings of this study cannot be generalized to the entire student population in China.

## Conclusion

The present study revealed the insufficient level of knowledge, moderate and acceptable level of practices, but high attitude toward foodborne diseases among these college students in western areas of China. Knowledge, attitudes, and practices varied based on demographic characteristics. To enhance knowledge, attitudes, and practices toward foodborne diseases, factors such as gender, nationality, education level, specialization type, and residence should be considered. Specifically, targeted courses or training on foodborne diseases should be increased for college students to improve their knowledge and practices for reducing foodborne diseases. Subsequent research could also employ qualitative methodologies to obtain a more profound insight into the factors that influence foodborne disease practices.

## Data Availability

The original contributions presented in the study are included in the article/Supplementary material, further inquiries can be directed to the corresponding author.
