# Supplementary material for: Knowledge, attitude, and practice toward foodborne disease among Chinese college students: a cross-sectional survey
Source: Front Public Health. 2024 Dec 17;12:1435486. doi: 10.3389/fpubh.2024.1435486 (PMC11685201; doi:10.3389/fpubh.2024.1435486)
Supplement: Supplementary file 1 [file Data_Sheet_1.pdf]

Supplementary material

Knowledge, attitude, and practice toward foodborne diseases among Chinese college students: a cross-sectional survey

Table S1 STROBE Statement

Table S2 Informed consent

Table S3 Questionnaire of KAP towards foodborne illnesses

Table S4 Frequency comparison of participants' knowledge about foodborne illnesses

Table S5 Frequency comparison of participants' attitude towards foodborne illnesses

Table S6 Frequency comparison of participants' behavior about foodborne illnesses

Table 1 STROBE Statement

Checklist of items that should be included in reports of ***cross-sectional studies***

Item No		Recommendation	Yes/No
**Title and abstract**	1	(*a*) Indicate the study's design with a commonly used term in the title or the abstract	Y
		(*b*) Provide in the abstract an informative and balanced summary of what was done and what was found	Y
**Introduction**			
Background/rationale	2	Explain the scientific background and rationale for the investigation being reported	Y
Objectives	3	State specific objectives, including any prespecified hypotheses	Y
**Methods**			
Study design	4	Present key elements of study design early in the paper	Y
Setting	5	Describe the setting, locations, and relevant dates, including periods of recruitment, exposure, follow-up, and data collection	Y
Participants	6	(*a*) Give the eligibility criteria, and the sources and methods of selection of participants	Y
Variables	7	Clearly define all outcomes, exposures, predictors, potential confounders, and effect modifiers. Give diagnostic criteria, if applicable	Y
Data sources/ measurement	8*	For each variable of interest, give sources of data and details of methods of assessment (measurement). Describe comparability of assessment methods if there is more than one group	Y
Bias	9	Describe any efforts to address potential sources of bias	Y
Study size	10	Explain how the study size was arrived at	Y
Quantitative variables	11	Explain how quantitative variables were handled in the analyses. If applicable, describe which groupings were chosen and why	Y
Statistical methods	12	(*a*) Describe all statistical methods, including those used to control for confounding	Y
		(*b*) Describe any methods used to examine subgroups and interactions	Y
		(*c*) Explain how missing data were addressed	Y

		(*d*) If applicable, describe analytical methods taking account of sampling strategy	N
		(*e*) Describe any sensitivity analyses	N
**Results**			
Participants	13*	(a) Report numbers of individuals at each stage of study—eg numbers potentially eligible, examined for eligibility, confirmed eligible, included in the study, completing follow-up, and analysed	Y
		(b) Give reasons for non-participation at each stage	Y
		(c) Consider use of a flow diagram	N
Descriptive data	14*	(a) Give characteristics of study participants (eg demographic, clinical, social) and information on exposures and potential confounders	Y
		(b) Indicate number of participants with missing data for each variable of interest	Y
Outcome data	15*	Report numbers of outcome events or summary measures	Y
Main results	16	(*a*) Give unadjusted estimates and, if applicable, confounder-adjusted estimates and their precision (eg, 95% confidence interval). Make clear which confounders were adjusted for and why they were included	Y
		(*b*) Report category boundaries when continuous variables were categorized	Y
		(*c*) If relevant, consider translating estimates of relative risk into absolute risk for a meaningful time period	N
Other analyses	17	Report other analyses done—eg analyses of subgroups and interactions, and sensitivity analyses	Y
**Discussion**			
Key results	18	Summarise key results with reference to study objectives	Y
Limitations	19	Discuss limitations of the study, taking into account sources of potential bias or imprecision. Discuss both direction and magnitude of any potential bias	Y
Interpretation	20	Give a cautious overall interpretation of results considering objectives, limitations, multiplicity of analyses, results from similar studies, and other relevant evidence	Y
Generalisability	21	Discuss the generalisability (external validity) of the study results	Y
**Other information**			
Funding	22	Give the source of funding and the role of the funders for	N

		the present study and, if applicable, for the original study on which the present article is based

*Give information separately for exposed and unexposed groups.

Note: An Explanation and Elaboration article discusses each checklist item and gives methodological background and published examples of transparent reporting. The STROBE checklist is best used in conjunction with this article (freely available on the Web sites of PLoS Medicine at http://www.plosmedicine.org/, Annals of Internal Medicine at http://www.annals.org/, and Epidemiology at http://www.epidem.com/). Information on the STROBE Initiative is available at www.strobe-statement.org.

Table S2 Informed consent

**Informed consent**
Dear students, we are inviting you to take a survey for research. This survey is completely voluntary. There are no negative consequences if you don't want to take it. If you start the survey, you can always change your mind and stop at any time.<br>**The purpose of this study:** We want to understand the levels of your knowledge, attitudes, and practices (KAP) toward foodborne diseases and to analyze the factors influencing practice actions.<br>**How to participate:** You will be invited to answer a series of questions related to the research topic. Surveys are usually conducted anonymously and your personal information including gender, age, nationality, educational level, monthly living expenses, type of specializations, residence, training and KAP will be kept confidential and will not be disclosed to any third party. The survey will take about 20 minutes.<br>**Risks and possible benefits:** Some questions may be personal or upsetting. You can quit the survey at any time. Surveys typically don't entail any risks, but they may consume some of your time. Participating in a survey offers you the chance to answer your knowledge, attitude, and practice actions on foodborne disease, contributing valuable information for food safety health education. Furthermore, de-identified data (all identifying information removed) may be shared with other researchers from the corresponding author upon reasonable request.<br>**Confidentiality and data security:** We will ensure the strict protection of your personal information to maintain its security and confidentiality. Your responses will be solely used for research purposes and will not be utilized for commercial use. The study results will be presented in an aggregated form and will not disclose any information that could personally identify you.<br>**Voluntarily to participate:** Your participation is completely voluntary, and you have the right to interrupt or withdraw from the survey at any time without any penalty or adverse effect. To take this survey, you must be at least 18 years old.<br>If you have any questions or need more information about this survey, please feel free to contact us. If you agree to take part in this survey, please sign below to confirm that you have read and understood the above and agree to participate in this survey.<br>Thank you for your cooperation and support!<br><br>Researcher signature Date:<br>Participant Signature: Date:

Table S3

Questionnaire of knowledge, attitude, and practice toward foodborne illnesses

Demographics of students

1. Gender:

①Male ②Woman

2. Age:

①＜20 year ②⩾20 year ③----- year

3. Ethnicity:

①The minority ②The Han nationality

4. Grade:

①Freshman ②Sophomore ③Junior ④Senior

5. Monthly living expenses:

①<1000 yuan ②1000~2000yuan ③>2000 yuan

6. Type of specializations:

①Non-medical field ②Medical field

7. Residence

①Cities and towns ②Rural area

8. Training of food safety

①Yes ②No

9. Onset of foodborne illnesses within 2 months

①Yes ②No

Investigation on knowledge about foodborne illnesses

(1) Foodborne diseases are caused by contamination of food and occur at any stage of the food production, delivery and consumption chain.

①True ②False

(2) Uncooked raw meat should be stored in the lower part of the refrigerator (-18℃ storage).

①True ②False

(3) Contact between cooked and uncooked foods can causes cross-contamination.

①True ②False

(4) Wearing gloves will reduce the food contamination.

①True ②False

(5) Contamination of foodstuffs cannot be detected using sense organs.

①True ②False

(6) Followed the correct method for washing food equipment.

①True ②False

(7) When preparing ingredients, it is customary to use the same knife to cut both vegetables and meat, which is easy to be contaminated.

①True ②False

(8) Contaminated foodstuffs always change their characteristics.

①True ②False

(9) Food contamination risk zone (5℃-60℃)

①True ②False

(10) Frequent food contact surface cleaning can prevent contamination of the food.

①True ②False

Investigation on attitude towards foodborne illnesses

(1) Covering the mouth during coughing and sneezing avoids contamination of food.

①Agree ②Neutral ③Disagree

(2) Do you prefer wearing a ring or watch inside the cafeteria.

①Agree ②Neutral ③Disagree

(3) Touching food manually without gloves induces food contamination.

①Agree ②Neutral ③Disagree

(4) After touching some parts of your body, you should wash yourself hands.

①Agree ②Neutral ③Disagree

(5) Raw food should be processed separately from cooked food.

①Agree ②Neutral ③Disagree

(6) Long and painted fingernails contaminate foodstuffs with pathogens.

①Agree ②Neutral ③Disagree

(7) Proper hygiene of the hand can prevent foodborne illnesses.

①Agree ②Neutral ③Disagree

(8) Pathogens can be sourced from food utensils.

①Agree ②Neutral ③Disagree

(9) You with abrasions on hand do not treat food ready.

①Agree ②Neutral ③Disagree

(10) Before jobs, the health condition of food handlers should be assessed.

①Agree ②Neutral ③Disagree

(11) To control foodborne diseases, it is necessary to maintain personal hygiene.

①Agree ②Neutral ③Disagree

(12) Hands are where most bacteria and microorganism originate.

①Agree ②Neutral ③Disagree

(13) Left cooked food for more than 2 hours from the refrigerator is unsafe.

①Agree ②Neutral ③Disagree

Investigation on behavior related to echinococcosis

(1) Do you wash your hands before handling and cooking food with soap and water.

①Never ②Sometimes ③Neutral ④Often ⑤Always

(2) Do you cover your hands with cut/sore before preparing food.

①Never ②Sometimes ③Neutral ④Often ⑤Always

(3) Do you wash your hands with soap and water after you touch the raw meat, poultry, and seafood.

①Never ②Sometimes ③Neutral ④Often ⑤Always

(4) Before using cooked food, you wash plates used for raw meat,poultry, and seafood.

①Never ②Sometimes ③Neutral ④Often ⑤Always

(5) Using the same cutting board for raw meat, poultry,seafood, and vegetables.

①Never ②Sometimes ③Neutral ④Often ⑤Always

(6) Using frozen meat thawed in the morning to cook in the evening.

①Never ②Sometimes ③Neutral ④Often ⑤Always

(7) Left cooked food near the counter, to be used the next day.

①Never ②Sometimes ③Neutral ④Often ⑤Always

(8) Store eggs at room temperature.

①Never ②Sometimes ③Neutral ④Often ⑤Always

(9) Using hot and soapy water after food preparation to disinfect countertops.

①Never ②Sometimes ③Neutral ④Often ⑤Always

(10) Using a thermometer to assess if the food is fully cooked.

①Never ②Sometimes ③Neutral ④Often ⑤Always

(11) Eating runny yolk eggs or items containing crude eggs.

①Never ②Sometimes ③Neutral ④Often ⑤Always

(12) After 3-4 days, should you discard refrigerated leftovers.

①Never ②Sometimes ③Neutral ④Often ⑤Always

(13) Check the production date and expiration date when you buy foodstuffs.

①Never ②Sometimes ③Neutral ④Often ⑤Always

(14) Clean your hands before cooking foodstuffs.

①Never ②Sometimes ③Neutral ④Often ⑤Always

(15) After handling dirty things, do you wash your hands?

①Never ②Sometimes ③Neutral ④Often ⑤Always

References: (1) Azanaw J et al. Food Safety Knowledge, attitude, and practice of college students, Ethiopia, 2019: a cross-sectional study. Biomed Res Int. 2021, 6686392. (2) Cai Z et al. Effect of WeChat-based intervention on food safety knowledge, attitudes and practices among university students in Chongqing, China: a quasi-experimental study. J Health Popul Nutr. 2023,42(1), 28. (3) Osaili TM, et al. Knowledge, practices, and risk perception associated with foodborne illnesses among females living in Dubai, United Arab Emirates. Foods. 2022,11(3), 290.

Table S4 Frequency comparison of participants' knowledge about foodborne illnesses

**Variables**	True	False	***p*-value**
**Gender**			
Male (182)	88%	12%	0.23
Female (263)	87%	13%	
**Age, years**			
18~20 years (313)	87%	13%	0.36
≥20 years (132)	88%	12%	
**Nationality**			
Ethnic minority (22)	89%	11%	0.50
Han Chinese (423)	88%	12%	
**Educational level**			
Lower-year undergraduate (297)	87%	13%	0.01
Upper-year undergraduate (148)	89%	11%	
**Monthly living expenses**			
<1000 yuan (16)	87%	13%	0.16
1000~2000 yuan (387)	88%	12%	
>2000 yuan (42)	85%	15%	
**Type of specializations**			
Non-medical field (351)	87%	13%	0.17
Medical field (94)	89%	11%	
**Residence**			
Cities and towns (157)	86%	14%	0.48
Rural area (288)	88%	12%	
**Training on foodborne diseases**			
Yes (41)	89%	11%	0.46
No (404)	88%	12%	

The Chi-square test was used to analyze the differences in categorical variables. Statistical significance was set at a level of $P \leq 0.05$. Lower-year undergraduate (freshman+ sophomore) and upper-year undergraduate (junior and senior).

Table S5 Frequency comparison of participants' attitude towards foodborne illnesses

**Variables**	Agree	Neutral	Disagree	***p*-value**
**Gender**				
Male (182)	81%	14%	5%	0.61
Female (263)	80%	15%	5%	
**Age, years**				
18~20 years (313)	80%	15%	5%	0.05
≥20 years (132)	82%	13%	5%	
**Nationality**				
Ethnic minority (22)	83%	11%	6%	0.27
Han Chinese (423)	81%	14%	5%	
**Educational level**				
Lower-year undergraduate (297)	80%	15%	5%	0.77
Upper-year undergraduate (148)	82%	13%	5%	
**Monthly living expenses**				
<1000 yuan (16)	80%	14%	6%	0.01
1000~2000 yuan (387)	80%	15%	5%	
>2000 yuan (42)	86%	10%	4%	
**Type of specializations**				
Non-medical field (351)	80%	15%	5%	0.04
Medical field (94)	83%	12%	5%	
**Residence**				
Cities and towns (157)	81%	13%	6%	0.02
Rural area (288)	81%	15%	4%	
**Training on foodborne diseases**				
Yes (41)	81%	14%	5%	<0.01
No (404)	78%	15%	7%	

The Chi-square test was used to analyze the differences in categorical variables. Statistical significance was set at a level of $P \leq 0.05$. Lower-year undergraduate (freshman+ sophomore) and upper-year undergraduate (junior and senior).

Table S6 Frequency comparison of participants' behavior about foodborne illnesses

Variables	Never	Sometimes	Neutral	Often	Always	*p*-value
**Gender**						
Male (182)	6%	9%	15%	26%	45%	<0.01
Female (263)	4%	9%	13%	27%	47%	
**Age, years**						
18~20 years (313)	5%	8%	14%	28%	45%	<0.01
≥20 years (132)	7%	10%	13%	22%	48%	
**Nationality**						
Ethnic minority (22)	4%	18%	16%	27%	35%	<0.01
Han Chinese (423)	5%	8%	13%	26%	47%	
**Educational level**						
Lower-year undergraduate (297)	5%	9%	14%	26%	45%	0.04
Upper-year undergraduate (148)	5%	9%	12%	26%	48%	
**Monthly living expenses**						
<1000 yuan (16)	3%	13%	14%	33%	38%	<0.01
1000~2000 yuan (387)	5%	9%	14%	26%	46%	

>2000 yuan (42)	4%	6%	10%	26%	54%	
**Type of specializations**						
Non-medical field (351)	6%	9%	14%	25%	46%	<0.01
Medical field (94)	3%	8%	11%	31%	47%	
**Residence**						
Cities and towns (157)	5%	7%	12%	24%	51%	<0.01
Rural area (288)	5%	10%	14%	27%	44%	
**Training on foodborne diseases**						
Yes (41)	7%	7%	12%	19%	54%	<0.01
No (404)	5%	9%	14%	27%	45%	

The Chi-square test was used to analyze the differences in categorical variables. Statistical significance was set at a level of $P \leq 0.05$. Lower-year undergraduate (freshman+ sophomore) and upper-year undergraduate (junior and senior).
